# Supplementary material for: Application of a Novel Formulation of 1-Aminocyclopropane-1-carboxylic Acid (ACC) to Increase the Anthocyanins Concentration in Table Grape Berries
Source: Plants (Basel). 2025 Mar 29;14(7):1058. doi: 10.3390/plants14071058 (PMC11990719; doi:10.3390/plants14071058)
Supplement: Supplementary file 1 [file plants-14-01058-s001.zip › plants-3529701-supplementary.pdf]

Supplementary Materials

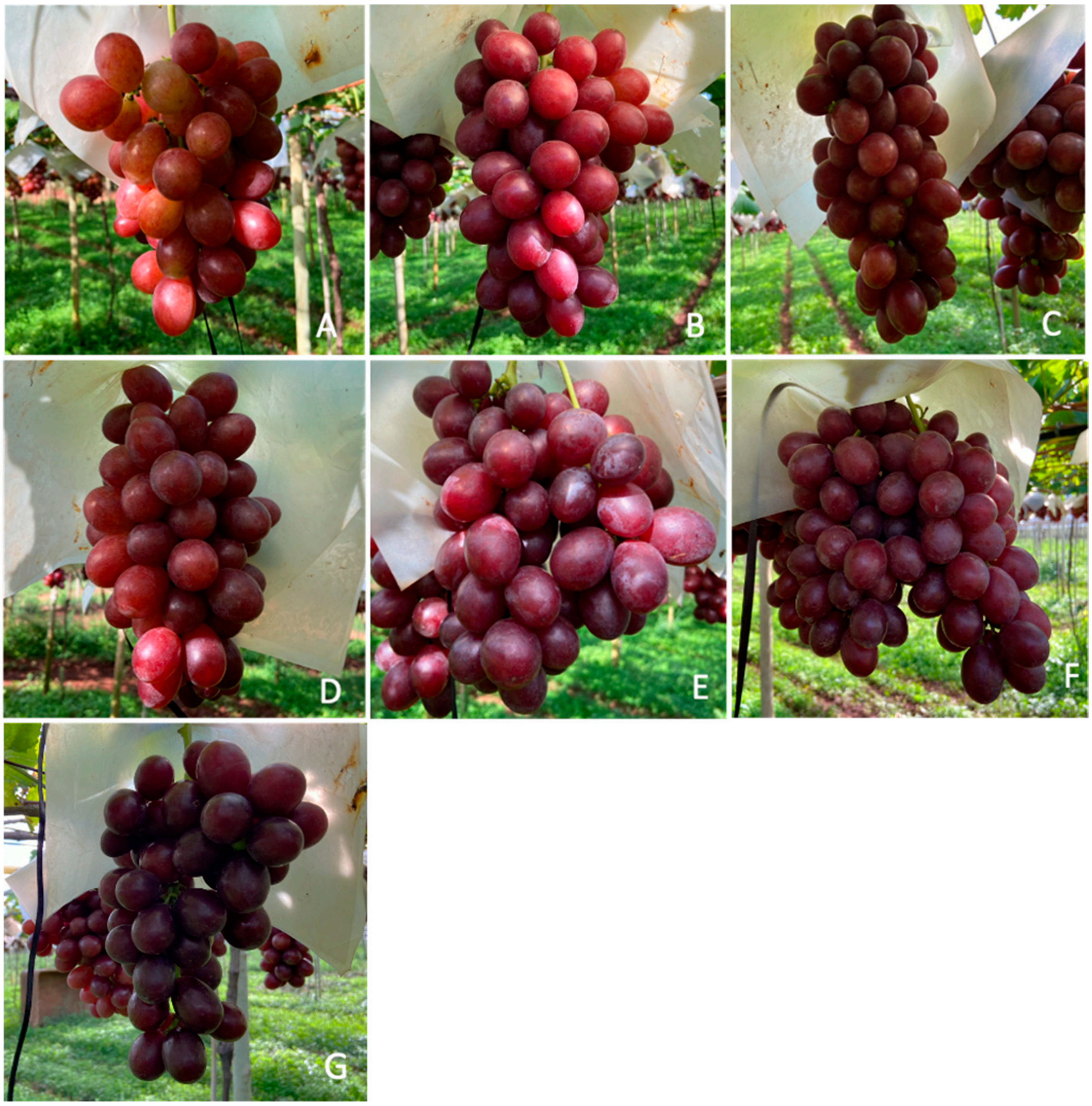

Figure S1. Bunches of 'Benitaka' table grape (*Vitis vinifera* L.) subjected to different treatments with with 1-aminocyclopropane-1-carboxylic acid – Accede® and abscisic acid – ProTone® applied to the entire canopy of the vines at *véraison.* Control (**A**); Accede® 25; 50; 75; 100; 125 g 100 L^{-1} (**B-F**); ProTone® 3.2 L ha^{-1} (**G**).

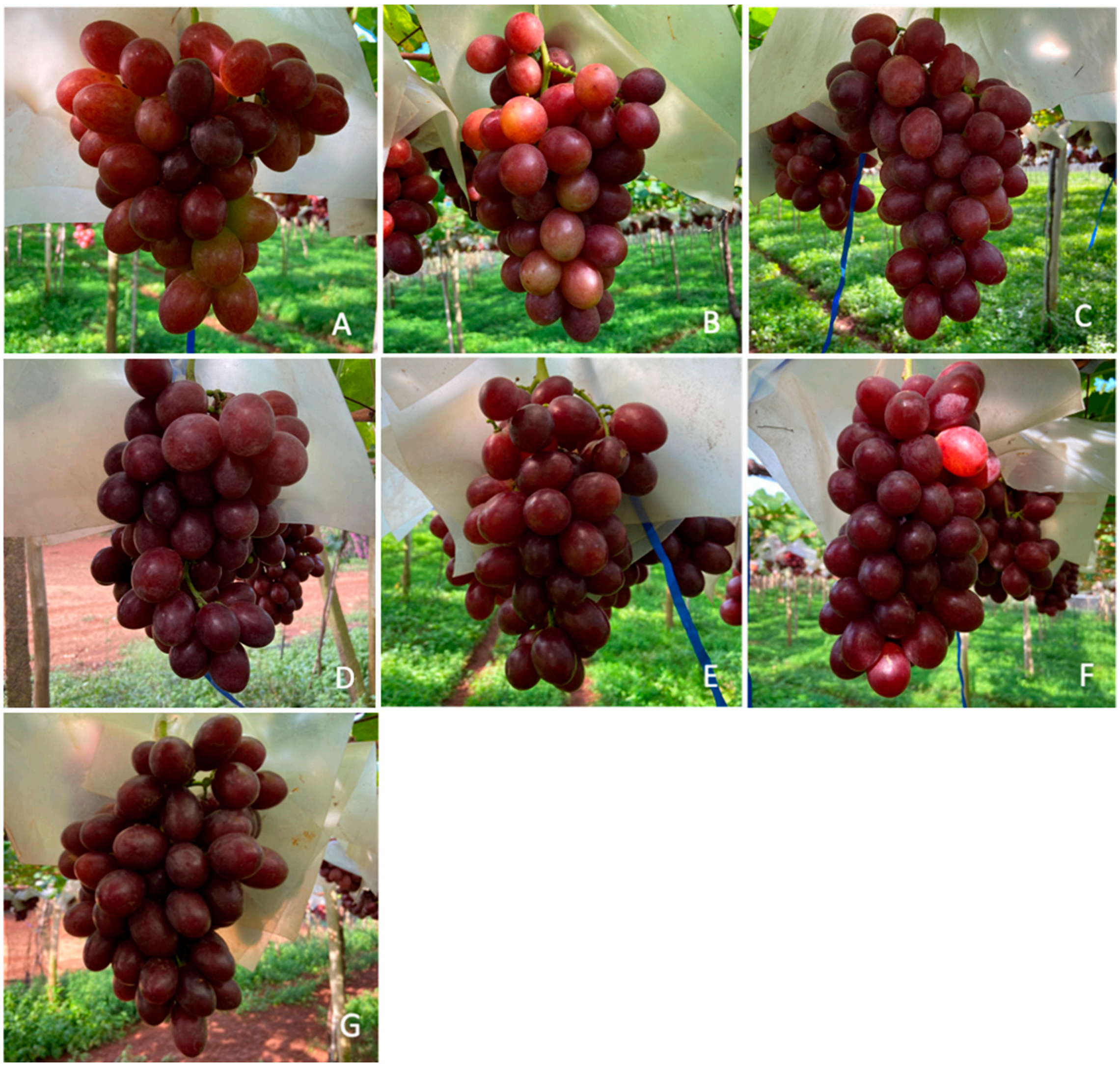

Figure S2. Bunches of 'Benitaka' table grape (*Vitis vinifera* L.) subjected to different treatments with 1-aminocyclopropane-1-carboxylic acid - Accede® and abscisic acid - ProTone® applied only to the bunches at *véraison*. Control (**A**); Accede® 25; 50; 75; 100; 125 g 100 L^{-1} (**B-F**); ProTone® 3.2 L ha^{-1} (**G**).
